# Liquid biopsy through non-blood fluids: The show must go on

**DOI:** 10.1016/j.jlb.2024.100272

**Published:** 2024-10-17

**Authors:** Angelo Dipasquale, Pasquale Pisapia, Carolina Reduzzi

**Affiliations:** IRCCS Humanitas Research Hospital, Milan, Italy; Young Committee, International Society of Liquid Biopsy, Spain; Young Committee, International Society of Liquid Biopsy, Spain; Department of Public Health, University of Naples “Federico II”, Naples, Italy; Young Committee, International Society of Liquid Biopsy, Spain; Department of Medicine, Weill Cornell Medicine, New York, USA

**Keywords:** Liquid biopsy, Solid tumors, Biological fluids

## Abstract

**Figure:**
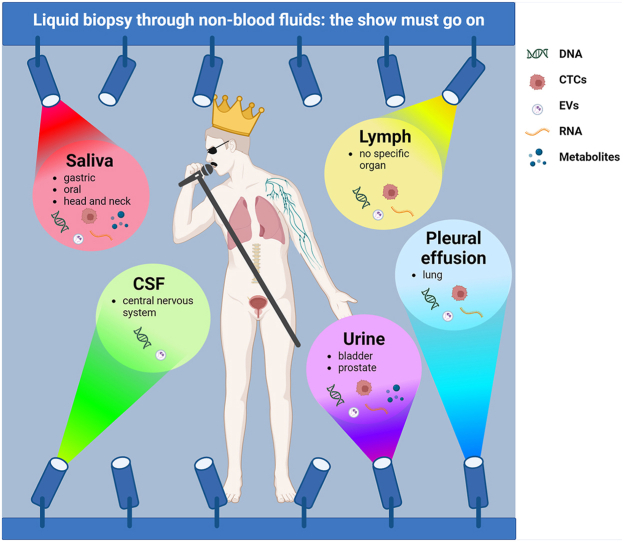

Liquid biopsy has significant revolutionized the management of cancer patients. In particular, the adoption of liquid biopsy as a complementary tool to tissue specimens is widely accepted in different clinical settings, from cancer interception to early detection, monitoring minimal residual disease and predict response and resistance to target treatments [1]. From a clinical point of view, the term liquid biopsy stands for circulating tumor DNA (ctDNA), a small fraction of the total cell free DNA (cfDNA) extracted from plasma samples. However, a number of different analytes, including circulating tumor cells (CTCs), circulating tumor RNA (ctRNA), extracellular vesicles (EVs), have to be taken into account to evaluate the complex molecular landscape of solid tumors [2]. In addition, the concept of liquid biopsy, which is most frequently associated to blood samples, can be extended to other body fluids, including cerebrospinal fluid (CSF), pleural effusion (PE), lymph, saliva and urine [3]. In particular, the adoption of tumor nucleic acids extracted from fluids more closely related to the metastatic site demonstrated a higher sensitivity than blood in detection of clinically relevant alterations for targeted treatments [4]. In this scenario, using different body fluids other than blood as well as the analysis of different analytes in addition to ctDNA may impact on diverse aspects of patient care, including cancer detection and screening, staging, prognostication, treatment selection, therapeutic response monitoring and early detection of treatment resistance or cancer recurrence [5].

Beyond body fluids, other source of tumor nucleic acids can be counted in the extended concept of liquid biopsy. Among these, a significant source of tumor related nucleic acids is represented by supernatant often discarded after cell pelleting for cytospin or cell block preparations [6]. In this setting, it has been highlighted a higher concordance between the molecular results obtained on cell free nucleic acids extracted from these fluids and matched tissue specimens [7]. Finally, another surrogate of liquid biopsy can be obtained from the collection of the fine needle aspiration (FNA) rinses, after preparing the smears for the microscopy evaluation, in a vial containing nuclease-free water [8]. This procedure is useful to obtain sufficient material for ancillary molecular studies while contemporary avoid to sacrifice precious and irreplaceable tissue material [8].

Several experiences outlined potential clinical validity and utility of non-blood fluids in the management of different cancers [[9], [10], [11], [12], [13], [14], [15], [16]]. A prospective experience including 74 patients with bladder cancer candidate to neoadjuvant chemotherapy and/or radical cystectomy analyzed urine cfDNA in urine samples at surgery [9]. Different molecular alterations including variant allele frequency and copy number variations were integrated in a machine learning model efficiently predicting pathological complete response (area under the curve, AUC, 0.80, p < 0.0001) and survival (hazard ratio, HR, 4.81, p 0.009) [9]. In perspective, this may help in properly selecting subjects needing consolidative post-operative therapies such as checkpoint inhibitors, to date showing conflicting results in an unselected population [10].

A cohort of 60 patients having human papilloma virus positive (HPV+) oropharyngeal carcinoma suitable for curative treatment showed high concordance between HPV + ctDNA in plasma and saliva samples (93 %) [11]. In matched longitudinal samples, ctDNA correlated with poor response to induction chemotherapy and/or residual disease after surgery [11]. A similar experience of 93 patients with HPV + oropharyngeal carcinoma found a significant impact of post-treatment salivary HPV + status in recurrence (HR 10.7, p 0.002) and survival (HR 25.9, p 0.002) [12]. Given this, liquid biopsy may be integrated in monitoring disease burden and residual disease in view of escalating/de-escalating strategies (salvage surgery, adjuvant (chemo)-radiation or lower-dose radiotherapy) [13].

Currently, diagnosis of adult-type diffuse gliomas (DGs) relies on presence of molecular markers and may be challenging for anatomical location and tumor heterogeneity. With blood being a poor source of ctDNA for the blood-brain barrier, CSF could provide more robust information for the proximity to brain parenchyma [14]. A pivotal experience prospectively assessing CSF-based liquid biopsy in 39 patients with newly diagnosed DGs used a polymerase chain reaction (PCR)-based panel testing isocitrate dehydrogenase (IDH) status and few other biomarkers, allowing a presumptive molecular diagnosis in 88.5 % of cases [15]. When appropriate, this may support differential diagnosis between IDH wild-type and IDH mutant gliomas, with obvious prognostic implication. A similar experience reported high sensitivity (96.5 %) in questioning H3K27M mutation in CSF of 24 patients with midline diffuse glioma [16]. With important issues in obtaining tissue from deep brain regions and with novel molecular drugs such as ONC201 showing promising antitumor activity in clinical trials, a non-invasive diagnosis for this entity becomes crucial [16].

Still, liquid biopsy analysis presents limitations that are common across all body fluids. These limitations consist mainly in (I) the rarity of tumor-derived analytes in the fluids, (II) the presence of non-tumor material, (III) the lack of universal markers for the identification of tumor-derived analytes and (IV) the need for a better standardization in preanalytical and analytical procedures. For these reasons, various technologies have been developed over the years to accurately detect and analyze the various tumor-derived circulating components.

Nonetheless, these technologies as well as the pre-analytical procedures associated to them have been developed and validated mainly in blood and might not show the same performance or not be the best solutions for other body fluids. For example, in saliva, the presence of microbial content and enzymes such as RNAses should be specifically addressed by developing methods able to identify and distinguish both tumor-derived and microbiota-derived biomarkers, while also assuring the preservation of circulating RNA [17]. Similarly, nucleases in urine constitute an issue due to their high activity in this fluid, in addition to high rate of bacterial contamination due to incorrect collection methods [18]. In the context of CSF, pre-analytical variables such as the tumor type/location and its proximity to the CSF space should be considered, in addition to those already factored in plasma processing (e.g. collection-to-processing time, volume, storage and DNA extraction methods) [19]. Moreover, it is important to consider that, conversely to other fluids, CSF is collected through a more invasive procedure (e.g. lumbar puncture) which might not be suitable for all patients and may lead to side effects [20].

Unfortunately, compared to blood, little effort in technological development has been employed so far. This is because, whereas blood-based liquid biopsy has a broad clinical applicability spanning the most common tumors (including breast, prostate, colorectal, and lung cancer), the use of alternative fluids is normally limited to specific tumors, such as prostate and bladder cancer for urine and brain tumors for CSF, reducing the interest of biotech/diagnostic companies and funding agencies in supporting the development and clinical validation of assays for specific non-blood body fluids. This leads to another limitation which is the current lack of solid clinical evidence which would require large-scale clinical studies and comprehensive validation of the results in independent cohorts, as was achieved for blood-based analytes such as CTCs and ctDNA.

Overall, current evidence of other body fluids in supporting liquid biopsy remains diversified and extremely promising. In our view, more efforts should be pursued to select properly clinical scenarios which may benefit from this non-blood tool, to overcome barriers related to funding and intrinsic methodological challenges and to validate clinical validity/utility within large and prospective studies. The show must go on.

## Declaration of competing interest

Authors declare to be members of the Young Committee of the International Society of Liquid Biopsy.

## Author's contribution

AD, PP, CR: conceptualization, methodology, software, validation, formal analysis, investigation, resources, data curation, Writing – Original Draft Preparation, Writing – Review & Editing. PP, CR: visualization, supervision, project administration.

## Ethical approval/Patient consent

Not required.

## Funding

The authors have not declared a specific grant for this review from any funding agency in the public, commercial or not-for-profit sectors.
